# From Electrical Morphology to Physiological Dynamics: Lessons From ECG Adoption for the Clinical Integration of Heart Rate Variability

**DOI:** 10.1111/eci.70249

**Published:** 2026-08-06

**Authors:** José Luis Puglisi, Gurpreet Sodhi, Samuel Minucci Carmargo, Daniel Gustavo Goroso

**Affiliations:** ^1^ College of Medicine California Northstate University Elk Grove California USA; ^2^ Center for Technological Research Universidade de Mogi das Cruzes Mogi das Cruzes São Paulo Brazil; ^3^ Instituto de Saúde, Secretaria de Estado da Saúde de São Paulo São Paulo São Paulo Brazil

**Keywords:** artificial intelligence, autonomic nervous system, cardiovascular monitoring, electrocardiography, heart rate variability, medical education

## Abstract

**Background:**

Heart rate variability (HRV) has remained a relatively finite and niche tool in cardiology despite decades of research supporting its physiological and clinical relevance. This limited adoption may resemble the early history of electrocardiography (ECG), which was initially regarded by many physicians as a laboratory instrument rather than a routine clinical tool. The delayed acceptance of ECG reflected technological limitations, cultural resistance and the need for clinicians to master unfamiliar concepts derived from physics and electrophysiology. HRV faces comparable barriers today. Although derived from ECG RR intervals, HRV requires interpretation of time‐domain, frequency‐domain, geometric and nonlinear indices that may appear mathematically complex and distant from conventional bedside reasoning.

**Aims:**

We argue that HRV should not be viewed as a replacement for ECG, but as an extension of ECG from electrical morphology to physiological dynamics.

**Materials and Methods:**

Lessons from ECG history were examined and compared with the current state of HRV adoption in clinical practice. The complementary diagnostic roles of ECG morphology and HRV analysis were considered, together with the potential contribution of wearable sensors, remote monitoring, artificial intelligence and large language models to facilitate HRV interpretation, education and clinical integration.

**Results:**

Whereas conventional ECG morphology identifies arrhythmias, conduction disturbances, ischemic alterations and overt electrical abnormalities, HRV provides insight into autonomic modulation, cardiovascular adaptability and systemic physiological regulation. The emergence of wearable sensors, remote monitoring, artificial intelligence and large language models creates an opportunity to overcome barriers that have limited HRV adoption. Artificial intelligence may serve as an educational and interpretive bridge, translating complex HRV metrics into clinically meaningful concepts while supporting medical training, artefact awareness, case‐based learning and workflow integration.

**Discussion:**

HRV faces barriers comparable to those encountered during the early adoption of ECG, including technological limitations, educational challenges and resistance to incorporating unfamiliar physiological concepts into routine clinical practice.

**Conclusion:**

Lessons from ECG history suggest that HRV adoption will depend not only on evidence but also on standardization, education, clinical interpretation and cultural acceptance within cardiology.

## Graphical abstract

Is heart rate variability today where electrocardiography was a century ago? Many of the barriers currently limiting the clinical adoption of HRV—technological complexity, lack of standardization, limited clinician training and interpretative uncertainty—closely resemble those encountered during the early years of the ECG. The historical evolution of ECG suggests that successful clinical adoption depends not only on scientific evidence but also on education, workflow integration, technological refinement and cultural acceptance.

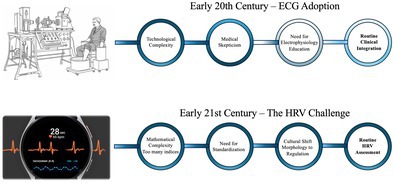

## 1 Introduction

Electrocardiography (ECG) remains one of the most important diagnostic technologies in modern medicine. Since its clinical introduction, the ECG has transformed cardiovascular evaluation by enabling the identification of arrhythmias, conduction disturbances, ischemic alterations and structural or electrophysiological disorders. More than a century after its introduction, the ECG remains a pillar of cardiology because of its accessibility, reproducibility, low cost and diagnostic utility.

Despite this success, conventional ECG interpretation remains centered on electrical morphology: waveforms, intervals, conduction pathways and rhythm classification. This provides essential information about cardiac electrical integrity but offers limited insight into the dynamic physiological regulation underlying cardiovascular function.

**FIGURE 1:**
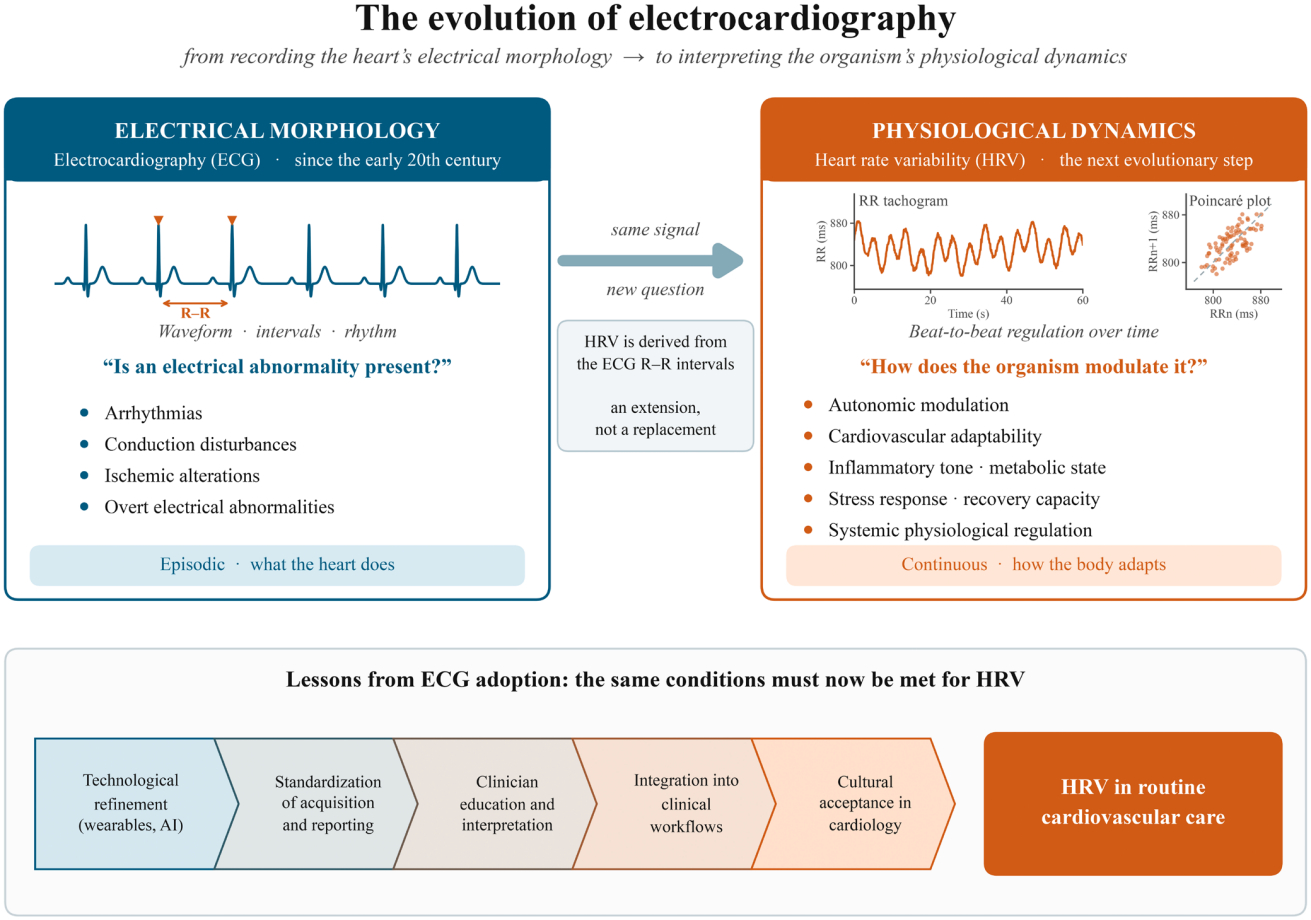
The evolution of electrocardiography: from recording the heart's electrical morphology to interpreting the organism's physiological dynamics. This schematic representation illustrates the conceptual transition from traditional electrocardiography to the clinical integration of heart rate variability (HRV). Electrical Morphology (Top Left): represents the classical ECG paradigm established in the early 20th century, which focuses on waveforms, intervals and rhythms to identify “what the heart does” and detect electrical abnormalities such as arrhythmias, conduction disturbances and ischemic alterations through episodic assessment. Physiological Dynamics (Top Right): Represents HRV as the next evolutionary step. Utilizing the same ECG RR‐intervals, it addresses a new question: “*how the organism modulates it?*” This approach provides a continuous analysis of “*how the body adapts*” through the study of autonomic modulation, cardiovascular adaptability and systemic physiological regulation using tools like the RR tachogram and Poincaré plot. The Transition (Center): emphasizes that HRV is derived from the same signal as the ECG and serves as a diagnostic extension rather than a replacement. Clinical Integration (Bottom): outlines the necessary stages for HRV to reach routine cardiovascular care, mirroring the historical adoption of the ECG. These include technological refinement (AI and wearables), standardization of reporting, clinician education, integration into workflows and cultural acceptance within the cardiology community.

Heart rate variability (HRV), derived from beat‐to‐beat fluctuations in RR‐intervals, provides a complementary perspective. HRV reflects the continuous interaction between sympathetic and parasympathetic modulation and may indirectly inform autonomic adaptability, cardiovascular regulation, inflammatory tone, metabolic state, sleep physiology, stress response and recovery capacity [1, 2, 3]. Rather than an isolated metric, HRV can be understood as an extension of electrocardiography itself: the ECG describes the electrical manifestation of cardiac activity, whereas HRV reflects the physiological dynamics regulating that activity over time. The ECG primarily asks whether electrical abnormalities are present; HRV explores how the organism modulates cardiac activity under changing physiological conditions. This distinction becomes increasingly relevant as cardiovascular medicine evolves from episodic diagnostic assessment toward continuous physiological monitoring (Figure 1).

## 2 Historical Lessons From ECG Adoption

The current marginal position of HRV in cardiology resembles the early trajectory of the ECG. When Einthoven introduced the electrocardiograph in the early twentieth century, it was not immediately embraced as a routine clinical tool. Early electrocardiographs were technically complex, physically large and initially regarded as laboratory instruments rather than bedside devices [4, 5]. Adoption required physicians to accept that invisible physiological phenomena could be measured and interpreted through unfamiliar physical principles—voltage, conduction, depolarization, repolarization and waveform morphology.

HRV faces an analogous challenge. Although derived from ECG recordings, its interpretation requires clinicians to grasp time‐domain indices, frequency‐domain power spectra, nonlinear dynamics, artefact correction, respiratory influences, recording duration and signal preprocessing [1, 2, 3, 6]—concepts that feel closer to biomedical engineering than traditional cardiology. The analogy is imperfect, but instructive: the ECG adoption was delayed not because it lacked clinical value, but because the medical community needed time, standardization, education and technological refinement to incorporate it into routine practice. HRV may be experiencing an analogous period of delayed translation.

## 3 Why HRV Remains a Niche Tool

Several converging barriers likely explain HRV's limited clinical adoption. The first is conceptual: HRV cannot be reduced to a single number. It comprises a family of metrics—SDNN, RMSSD, pNN50, LF, HF, VLF, the LF/HF ratio, the triangular index, entropy, detrended fluctuation analysis and other nonlinear indices [1]—each capturing a different facet of autonomic regulation, which clinicians without specific training may find disjointed.

The second is methodological. HRV interpretation depends heavily on recording context: duration, body position, respiration, sleep state, activity, sensor type, ectopy, artefact removal, medications, age, sex and disease status [3, 6]. The 1996 Task Force standards provided a foundation, but implementation across clinical settings remains inconsistent [1].

The third is cultural. Cardiology has traditionally prioritized anatomical lesions, ischemia, arrhythmias and hard endpoints. HRV instead asks clinicians to think in terms of regulation, adaptability and dynamic instability—a different conceptual framework from morphology‐based reasoning.

The fourth is translational. Unlike ST‐segment elevation, which carries a universally recognized, guideline‐backed meaning and triggers immediate action, HRV parameters lack standardized, evidence‐based cutoffs and specific diagnostic algorithms. An abnormal SDNN or RMSSD does not yet translate into a clear clinical directive. HRV has shown prognostic relevance after myocardial infarction and in heart failure, but its positive predictive value may be limited when used in isolation [7, 8, 9, 10]. It may be more valuable as a longitudinal biomarker of residual physiological instability and recovery than as a single measurement, and it is best integrated with other clinical, imaging and biochemical data rather than used alone.

Finally, there is a workflow barrier: clinicians need practical interpretation pathways rather than raw tachograms or spectral plots, which requires validated algorithms, quality control and decision‐support tools. Together, these barriers closely resemble the challenges of early ECG adoption, suggesting HRV may be at a comparable stage of its clinical trajectory.

## 4 HRV As Physiological Extension, Not Replacement, of ECG

HRV should not be framed as a replacement for conventional ECG, whose diagnostic value for atrial fibrillation, atrioventricular block, bundle branch block, ischemia, QT prolongation and ventricular arrhythmias remains indisputable. Instead, HRV adds a functional layer to ECG interpretation, shifting cardiovascular assessment from a purely morphological question to a physiological one. This distinction is particularly relevant in heart failure, where patients may show persistent autonomic dysregulation despite stable ECG morphology or optimized therapy and in the era of wearables, smartwatches and remote monitoring, which allow longitudinal observation across sleep, activity, stress, illness and recovery rather than short, controlled snapshots. Because autonomic regulation is interconnected with inflammatory, respiratory, metabolic and neurophysiological processes, HRV may hold relevance not only for cardiology but also for preventive medicine, rehabilitation, sports physiology, intensive care and digital health.

A frequent criticism is that HRV yields physiological information without translating into clinical decisions. Yet this may overlook the specific value of physiological biomarkers: revealing dysregulation before overt structural or electrical abnormalities appear. Traditional risk assessment relies on anatomical, electrical, imaging and biochemical findings that often represent advanced disease; physiological dysregulation may emerge earlier, reflecting altered autonomic control and adaptive capacity. Reduced HRV may reflect a loss of regulatory flexibility or persistent autonomic imbalance associated with adverse outcomes, particularly when assessed longitudinally. After myocardial infarction, clinically stable patients may still show impaired autonomic recovery not captured by ECG morphology; in heart failure, HRV may complement biomarkers such as NT‐proBNP in identifying early decompensation. Rather than a single diagnostic metric, HRV may thus contribute to dynamic cardiovascular risk stratification, revealing progressive deterioration or residual instability before clinical events become manifest.

The integration of electrophysiology, autonomic neuroscience and digital medicine (artificial intelligence, wearables, longitudinal analytics) may ultimately support a broader framework of continuous physiological surveillance. Continuous HRV assessment could transform cardiovascular evaluation from episodic observation into longitudinal surveillance, enabling a more proactive, personalized approach, provided it remains integrated with ECG, imaging, biomarkers and clinical assessment rather than used as a standalone test.

## 5 The Role of Artificial Intelligence

**FIGURE 2:**
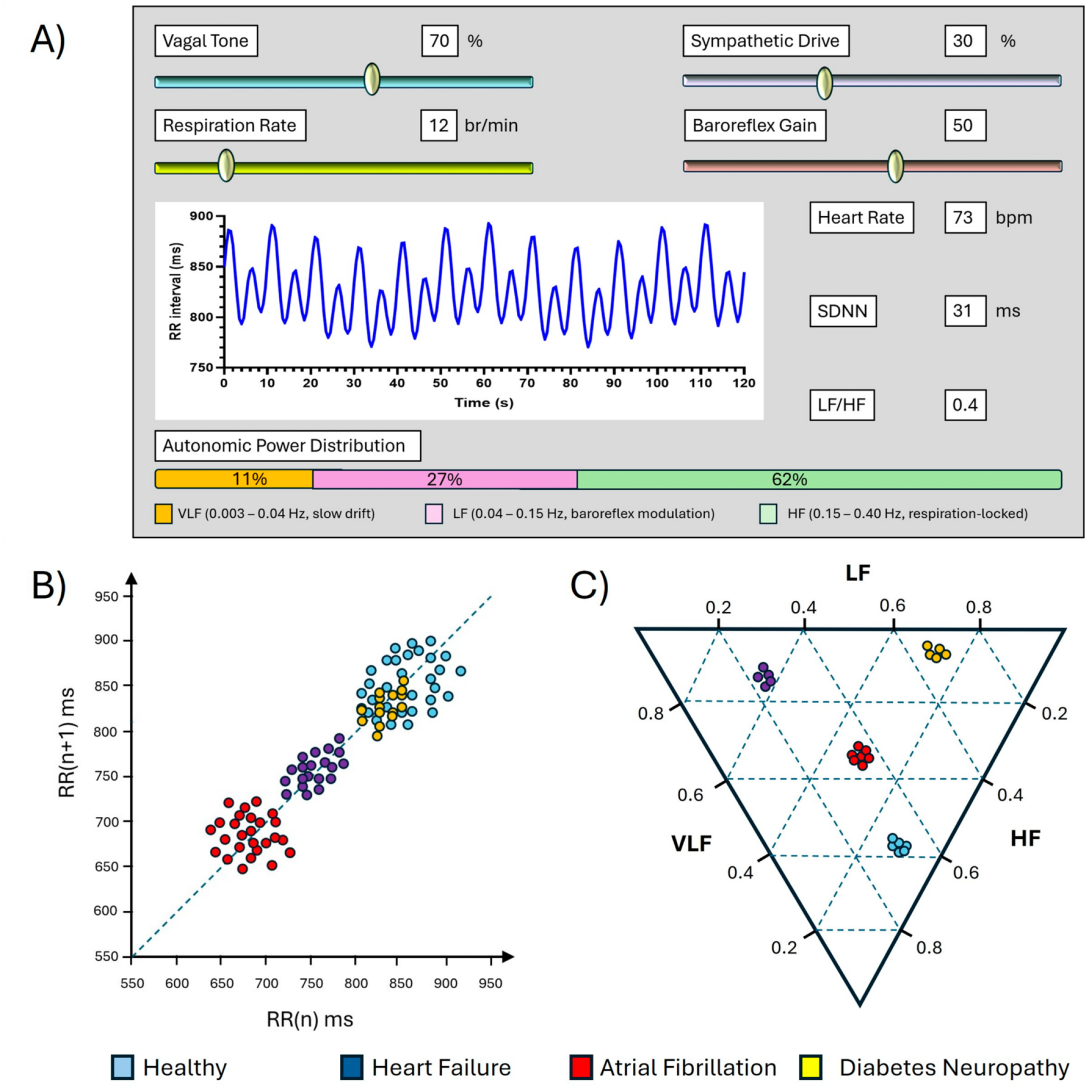
Interactive AI‐based tools for teaching heart rate variability (HRV). (A) *Autonomic feedback‐loop simulator*: A real‐time interface in which four independent physiological drivers—vagal tone, sympathetic drive, respiration rate and baroreflex gain—can be adjusted by the user (shown here at illustrative resting values: vagal tone 70%, sympathetic drive 30%, respiration 12 breaths/min, baroreflex gain 50). Adjusting these inputs regenerates the simulated RR‐interval tachogram (plotted against elapsed time, 0–120 s) and updates derived outputs, heart rate, SDNN and the LF/HF ratio, together with the relative VLF/LF/HF power distribution, in real time, allowing trainees to observe directly how a given autonomic state shapes both the time‐domain signal and its underlying spectral composition. (B) *Poincaré plot* (RR(n) vs. RR(*n* + 1)) superimposing four simulated physiological patterns (colour key as in C): Short‐term, beat‐to‐beat variability (SD1, dispersion perpendicular to the identity line) and longer‐term variability (SD2, dispersion along the identity line) jointly separate healthy recordings, which retain the broadest dispersion, from the progressively tighter, less variable clusters characteristic of heart failure, diabetic autonomic neuropathy and atrial fibrillation. (C) *Autonomic Power Triangle*: A ternary plot of relative VLF, LF and HF spectral power (axis labels alongside their respective edges; gridlines at intervals of 0.2), using the same simulated patterns and colour scheme as in (B). Each pattern occupies a distinct region: heart failure clusters toward the VLF corner, diabetic neuropathy toward the LF corner, atrial fibrillation near the centroid, and healthy recordings toward the HF corner, illustrating that autonomic dysregulation is better characterized by the overall balance among all three frequency bands than by any single band in isolation. All data shown in (A)–(C) are simulated for illustrative and educational purposes and do not represent measured patient recordings.

Artificial intelligence may help accelerate HRV adoption by narrowing the educational and interpretive distance between complex metrics and clinical reasoning, not as an autonomous diagnostic authority, but as an educational, translational and decision‐support tool. Beyond the classical textbook or video classes, now AI can provide simulations or novel graphical representations with multidimensional plots. An interactive physiological simulator (Figure 2A) can let trainees manipulate vagal tone, sympathetic drive, respiration rate and baroreflex gain and watch, in real time, how these drivers reshape the RR tachogram and the resulting VLF, LF and HF power distribution, making explicit, for example, why the HF band tracks respiration and why the LF cannot be read as a pure sympathetic index once respiration slows into the LF range. Student that are more comfortable with inductive approach can construct the generalization (“HF tracks respiration,” “LF depends more on baroreflex than pure sympathetic tone”) from accumulated observations. By the same token, deductive students can be told “vagal blockade removes parasympathetic input to the SA node; predict what happens to HF power” and then run the simulation to check their prediction. Implementing the predict‐observe‐explain (POE) educational technique: state the principle, derive the expected outcome, then use the simulation as the “experiment” that confirms or falsifies the prediction.

AI can also generate Poincare plots (Figure 2B) for healthy and pathological cases. The student learns to recognize that a *comet shape* means healthy modulation, a *compact ball* means autonomic collapse, and a *diffuse fan* means chaotic conduction by seeing enough examples that the shape‐to‐category mapping becomes perceptual rather than calculated. This is the same mechanism behind ECG pattern recognition training or radiological diagnosis: repeated, feedback‐guided exposure to labelled examples builds what the perceptual learning literature calls perceptual expertise—fast, largely nonverbal category recognition. It turns turn otherwise abstract nonlinear metrics into recognizable, comparable shapes.

Finally, a new type of diagram like the *Autonomic Power Triangle* (Figure 2C) materializes three otherwise abstract quantities, VLF, LF and HF power, into a single, concrete geometric location for each patient, so that autonomic balance becomes something a student can see and compare spatially rather than reconstruct mentally from three disconnected percentages. Disease‐associated shifts in spectral balance are no longer abstract numerical differences but visible movement within a fixed frame, anchoring the concept of autonomic regulation to a specific, memorable place in the triangle. Finally, AI‐assisted tools should be developed transparently, with quality indicators, explainable outputs, uncertainty estimates and clinician‐facing interpretations that support, rather than replace, physiological reasoning.

## 6 Practical Recommendations for Clinical Integration

The history of ECG shows that adoption of a new technology requires more than technical validity; it requires cultural transformation, achieved only once instruments became practical, interpretation was standardized, clinicians were educated, and the signal was embedded into diagnostic workflows. HRV now stands at a comparable position. The transition toward predictive, preventive, personalized and participatory medicine [11, 12], driven by artificial intelligence, wearables, remote monitoring and individualized analytics, creates favourable conditions for its adoption, but the same risks remain: overinterpretation, inconsistent methodology and insufficient clinician education. HRV should therefore be introduced cautiously and systematically. Moving it from a niche research metric toward broader clinical relevance requires several concrete steps.

HRV education should be incorporated into medical, cardiology and digital‐health curricula, combining case libraries that demonstrate early physiological detection (post‐myocardial infarction risk stratification, heart failure monitoring, diabetic autonomic neuropathy, critical illness and post‐viral autonomic dysfunction) with deductive problem sets that require deriving indices and predicting their behaviour from autonomic principles, allowing AI‐assisted tools to adapt to inductive and deductive learning preferences within the same content base. Reports should be simplified without being oversimplified, indicating recording duration, acquisition device, rhythm quality, artefact burden, posture and whether interpretation is based on short‐ or long‐term data. Clinical workflows should define further validated use cases, including postural intolerance, rehabilitation and sleep and recovery assessment and longitudinal autonomic monitoring may help identify deterioration in hospitalized patients. HRV should be integrated with other data streams (symptoms, ECG, imaging, biomarkers, medications and longitudinal trends) rather than interpreted alone.

## 7 Conclusion

HRV may represent the next evolutionary step of electrocardiography: from recording the heart's electrical morphology to interpreting the organism's physiological dynamics. Its limited clinical adoption may not reflect a lack of relevance but a familiar historical pattern; ECG itself was once considered technically complex, culturally disruptive and impractical for routine use until technological refinement, standardization, education and clinical integration made it indispensable. Wearable technologies and artificial intelligence now offer an opportunity to accelerate an analogous transformation for HRV, but success will depend on rigorous methodology, careful interpretation, clinician education and integration into meaningful workflows. HRV should not be framed as a replacement for ECG, but as its physiological expansion: ECG shows the electrical events of the heart; HRV helps reveal how the organism regulates those events over time.

## Funding

This work was supported by FAPESP—São Paulo Research Foundation (Grant 2021/14231‐0). The authors acknowledge the financial support provided by the Coordination for the Improvement of Higher Education Personnel (CAPES) under its institutional publishing agreement (ordinance no. 120 of April 26, 2024). The funding organizations had no role in the design, preparation, or conclusions of this manuscript.

## Conflicts of Interest

The authors declare no conflicts of interest.

## Data Availability

The datasets generated during and/or analysed during the current study are available from the corresponding author on reasonable request.

## References

[1] Task Force of the European Society of Cardiology and the North American Society for Pacing and Electrophysiology , “Heart Rate Variability: Standards of Measurement, Physiological Interpretation, and Clinical Use,” Circulation 93, no. 5 (1996): 1043–1065. 8598068

[2] F. Shaffer and J. P. Ginsberg , “An Overview of Heart Rate Variability Metrics and Norms,” Frontiers in Public Health 5 (2017): 258, 10.3389/fpubh.2017.00258. 29034226 PMC5624990

[3] R. E. Kleiger , P. K. Stein , and J. T. Bigger, Jr. , “Heart Rate Variability: Measurement and Clinical Utility,” Annals of Noninvasive Electrocardiology 10, no. 1 (2005): 88–101, 10.1111/j.1542-474X.2005.10101.x. 15649244 PMC6932537

[4] M. Rivera‐Ruiz , C. Cajavilca , and J. Varon , “Einthoven's String Galvanometer: The First Electrocardiograph,” Texas Heart Institute Journal 35, no. 2 (2008): 174–178. 18612490 PMC2435435

[5] B. Grob , “Willem Einthoven and the Development of the String Galvanometer: How an Instrument Escaped the Laboratory,” History and Technology 22, no. 4 (2006): 369–390, 10.1080/07341510601003081.

[6] M. P. Tarvainen , J. P. Niskanen , J. A. Lipponen , P. O. Ranta‐aho , and P. A. Karjalainen , “Kubios HRV—Heart Rate Variability Analysis Software,” Computer Methods and Programs in Biomedicine 113, no. 1 (2014): 210–220, 10.1016/j.cmpb.2013.07.024. 24054542

[7] E. H. Hon and S. T. Lee , “Electronic Evaluation of the Fetal Heart Rate. VIII. Patterns Preceding Fetal Death, Further Observations,” American Journal of Obstetrics and Gynecology 87 (1963): 814–826. 14085784

[8] M. M. Wolf , G. A. Varigos , D. Hunt , and J. G. Sloman , “Sinus Arrhythmia in Acute Myocardial Infarction,” Medical Journal of Australia 2, no. 2 (1978): 52–53. 713911 10.5694/j.1326-5377.1978.tb131339.x

[9] M. T. La Rovere , J. T. Bigger, Jr. , F. I. Marcus , A. Mortara , P. J. Schwartz , and ATRAMI Investigators , “Baroreflex Sensitivity and Heart‐Rate Variability in Prediction of Total Cardiac Mortality After Myocardial Infarction,” Lancet 351, no. 9101 (1998): 478–484, 10.1016/S0140-6736(97)11144-8. 9482439

[10] J. Sztajzel , “Heart Rate Variability: A Noninvasive Electrocardiographic Method to Measure the Autonomic Nervous System,” Swiss Medical Weekly 134, no. 35–36 (2004): 514–522. 15517504 10.4414/smw.2004.10321

[11] P. Lee , C. Goldberg , and I. Kohane , The AI Revolution in Medicine: GPT‐4 and Beyond (Pearson Education, 2023).

[12] J. Denny and F. Collins , “Precision Medicine in 2030—Seven Ways to Transform Healthcare,” Cell 184, no. 6 (2021): 1415–1419, 10.1016/j.cell.2021.01.015. 33740447 PMC9616629

